# Effect of Exercise Training on Physical Fitness Among Young Tennis Players: A Systematic Review

**DOI:** 10.3389/fpubh.2022.843021

**Published:** 2022-03-02

**Authors:** Wensheng Xiao, Soh Kim Geok, Xiaorong Bai, Te Bu, Mohd Rozilee Norjali Wazir, Othman Talib, Wenfang Liu, Chongjiang Zhan

**Affiliations:** ^1^Department of Sports Studies, Faculty of Educational Studies, Universiti Putra Malaysia, Selangor, Malaysia; ^2^Department of Sports Studies, Faculty of Education Studies, Hunan Normal University, Changsha, China; ^3^Department of Science and Technical Education, Faculty of Educational Studies, Universiti Putra Malaysia, Selangor, Malaysia; ^4^Department of Sports Studies, Graduate School, Adamson University, Manila, Philippines; ^5^Sports Military Training Department, Jiyang College, Zhejiang Agriculture & Forestry University, Zhuji, China

**Keywords:** speed, strength, power, endurance, agility, flexibility, physical fitness, exercise training

## Abstract

**Background:**

Physical fitness comprises both health- and skill-related components that have been shown to correlate with the competitive ability of the athletes. Competitive ability of the athletes is strongly related to the positive or poor characteristics of physical fitness. Additionally, the adolescent stage is critical for the development of physical fitness. Physical fitness training for young tennis players should receive more attention. However, the current literature is deficient in in-depth reviews of the effects of exercise training on the physical fitness of young tennis players.

**Objective:**

This review is aimed to investigate the effects of exercise training on physical fitness among young tennis players.

**Methods:**

From October 2020, a comprehensive search was undertaken in four electronic databases (SCOPUS, PubMed, EBSCOhost (SPORTDiscus), and CINAHL Plus) and also on Google Scholar and other sources of gray literature references. The methodological quality of included studies was assessed using the Physiotherapy Evidence Database scale and the over scientific evidence was determined using the best evidence synthesis (BES). This review included only studies that employed an experimental design to assess the physical fitness components of young tennis players.

**Results:**

Nine articles on exercise training met all inclusion criteria and were included in this systematic review. The studies were of a high standard of quality. The research findings are relatively credible. The results indicated that speed (*n* = 8) and agility (*n* = 8) were the most often investigated performance characteristics in exercise training interventions with young tennis players, followed by power (*n* = 7), strength (*n* = 4), and flexibility (*n* = 1). Exercise training significantly increased the physical fitness of young tennis players in terms of speed and agility. There is a lack of evidence about strength and flexibility. Meanwhile, there is conflicting evidence regarding the effect on power, and yet there is no evidence regarding the effect of exercise training on endurance.

**Conclusions:**

This systematic review established a compelling case for the beneficial effects of exercise training interventions on physical fitness in youngtennis players. The review identifies current research gaps (i.e., athlete gender, with a particular emphasis on female athletes) that should be addressed in future experimental studies.

**Systematic Review Registration:**

https://www.crd.york.ac.uk/prospero, identifier CRD 42020213145.

## Introduction

Tennis is the world's second most popular sport, trailing only soccer. It is played in 195 countries and has an estimated 87 million fans (who have played tennis at least once) and represents 1.17% of the world's population (1, 2). Tennis has evolved from a predominantly technical sport in which sport-specific technical skills (e.g., stroke skills) predominated to a more explosive sport characterized by increasing serve and stroke velocity and requiring significantly increased physical demands (3–5). Physical fitness levels of the tennis players are critical in determining who wins and who loses, especially those with extremely close competitive levels (6, 7). Tennis players must possess a combination of agility, speed, strength, aerobic capacity, and other physical fitness components in order to execute advanced shots and compete well against increasingly competent opponents (4, 5). This extraordinary performance cannot be boiled down to a single distinguishing physical attribute. Tennis demands a delicate interplay of several components of physical fitness. Tennis skill development and performance are the procedures that underpin these components of physical fitness (5). A healthy physical structure is critical for an athlete's performance to improve (8). Additionally, the International Tennis Federation recommended that tennis players undergo a physical fitness examination. It considers that speed, strength, flexibility, agility, and endurance are all comprehensive measures of a tennis player's physical fitness (9). As a result, these specific components of physical fitness were chosen for this study.

It is worth noting that adolescence is the most critical stage of human growth and development. Studies have established that adolescence is a critical period for tennis players' physical fitness development (10–12), as physical fitness is one of the most important factors affecting the competitive ability of the athletes (13, 14). As a result, it may be instructive to evaluate the effects of various training approaches on the physical fitness of young tennis players. Chen et al. (15) found that proper exercise training can significantly increase the general fitness of young tennis athletes. However, to our knowledge, no systematic examination of the specific benefits of exercise training on the physical fitness components of young tennis players has been conducted yet. Therefore, this systematic review's objective is to evaluate whether exercise training enhances the physical fitness component of young tennis players.

## Materials and Methods

### Protocol and Registration

The protocol for this systematic review followed the Preferred Reporting Items for Systematic Reviews and Meta-Analyses guidelines (16), and it was prospectively registered in the International Prospective Register of Systematic Reviews: https://www.crd.york.ac.uk/prospero, CRD 42020213145.

### Search Strategy

SCOPUS, PubMed, EBSCOhost (SPORTDiscus), and CINAHL Plus were used to conduct a comprehensive search of the literature. The search lasted from the commencement date to October 2020. A search was performed using the following combination of keywords from each database: (“exercise training^*^” OR “fitness training^*^” OR “physical training^*^” OR “physical activity^*^” OR “physical exercise^*^” OR “physical therapy” OR “exercise^*^” OR “fitness” OR “rehabilitation” OR “aerobic exercise^*^” OR “functional performance” OR “exercise therapy” OR “physical intervention^*^” OR “physical rehabilitation^*^” OR “motor activity^*^” OR “motor skill intervention^*^” OR “active play” OR “active game^*^”) AND (“physical fitness” OR “physical endurance” OR “cardiorespiratory fitness” OR “physical conditioning” OR “fitness, physical” OR “speed” OR “power” OR “strength” OR “endurance” OR “flexibility” OR “agility”) AND (“adolescent tennis player^*^” OR “juvenile tennis player^*^” OR “teenager tennis player^*^” OR “youth tennis player^*^” OR “young tennis player^*^” OR “junior tennis player^*^”). Additionally, supplementary searches were conducted using Google Scholar. To ensure that the retrieval of publications was as extensive as possible, we selected eligible studies for inclusion from prior meta-analyses.

### Eligibility Criteria

Population, intervention, comparison, outcome, and study design (PICOS) was used to identify the studies included in this study (Table 1). Studies were included if they met the following criteria: (1) the selected study must be a peer-reviewed publication written in English that discusses a randomized controlled trial (RCT) or a non-RCT (non-RCT) that assessed the effect of exercise training interventions on overall physical fitness; (2) participants of the study must be young tennis players (male and female); (3) this study may include any exercise training method on physical fitness components. Studies that employ one or a combination of two or more types of exercise training methods as interventions are also included; (4) this study examined the effect of exercise training (i.e., physical exercise, physical therapy, exercise, fitness, rehabilitation, and aerobics) on young tennis players and evaluated at least one physical fitness component outcome, such as speed, strength, power, endurance, agility, and flexibility.

**Table 1:** Inclusion criteria based on PICOS (population, intervention, comparison, outcome, and study design).

**PICOS**	**Detail**
Population	Young tennis players (male/female, age 12–18 years old)
Intervention	Exercise training
Comparison	Multiple and single-group trials
Outcome	Physical fitness components (speed, power, strength, endurance, flexibility, agility)
Study designs	RCT or Non-RCT

*RCT, randomized controlled trial*.

Studies were excluded if: (1) those that included young athletes from other sports; (2) those that combined exercise training interventions with other non-exercise training and included unsupervised training sessions; (3) observational studies and interventions focused exclusively on counseling for exercise training implementation were excluded; and (4) articles published and unpublished in languages other than English, conference abstracts, and letters to the editor, case reports, and brief communications were excluded.

### Study Selection

**Figure 1:**
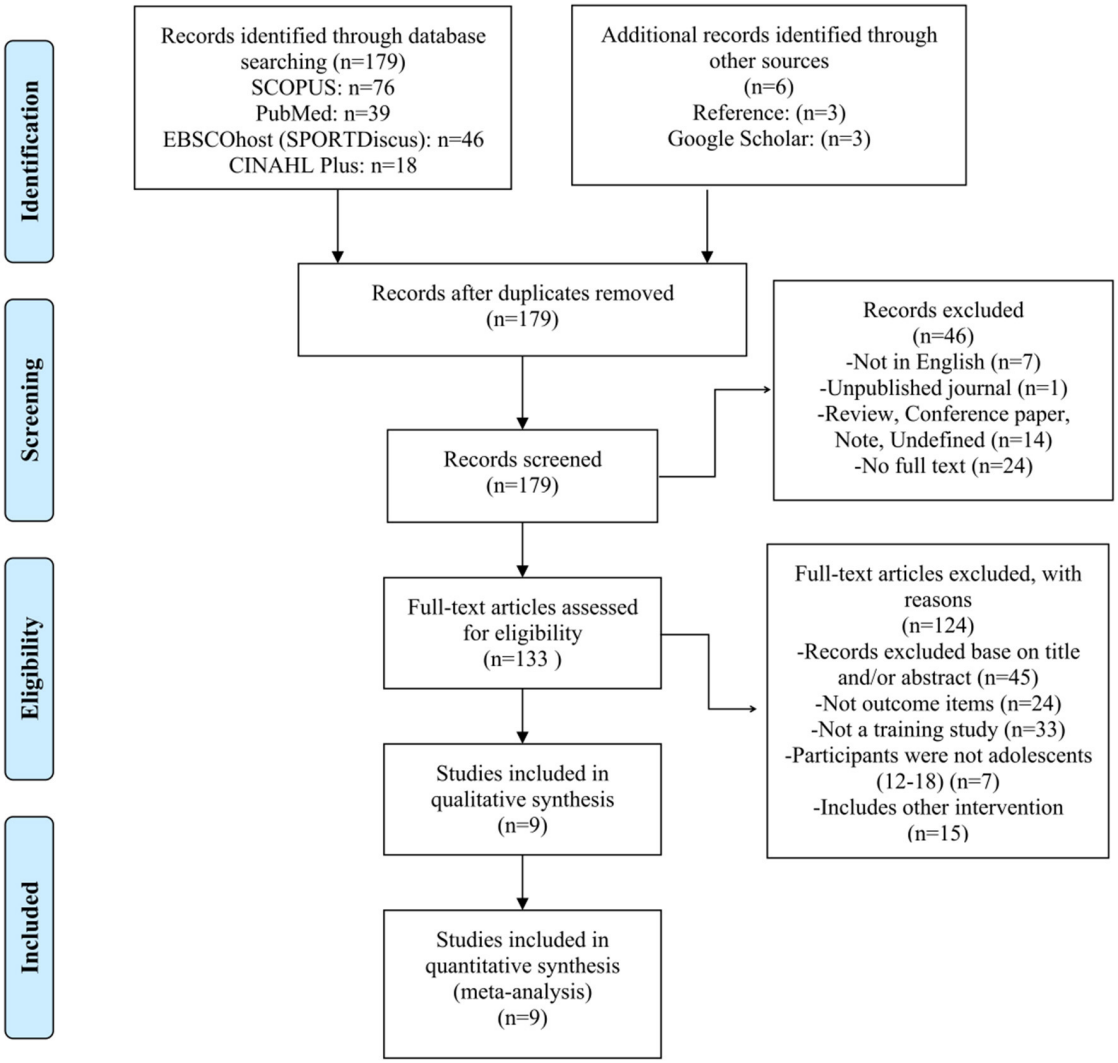
The Preferred Reporting Items for Systematic Reviews and Meta-Analyses (PRISMA) flow diagram of the literature selection process.

After conducting a search in four international electronic databases, the information about the retrieved studies (i.e., title, author, and year) was uploaded into Mendeley reference management software to eliminate duplicates. To begin, this study benefited from the assistance of an experienced librarian during the retrieval process. Second, two independent reviewers screened the title and abstract for suitable full-text studies. Then they examined the entire text of the studies in accordance with the criteria for inclusion and exclusion and reached a decision based on the literature into the research standard. Throughout this process, the two independent reviewers (Xiao and Bai) worked independently. If a disagreement arose, a third reviewer (Soh) was consulted until consensus was obtained. The details of the selecting procedure are depicted in Figure 1.

### Data Extraction and Quality Assessment

After completing the research selection process, two independent reviewers (Xiao and Bai) gathered pertinent data from each included study, such as the study title, participant characteristics, study design, specifics of the exercise training program and index, and study outcomes.

The methodology of the included studies was evaluated separately by two reviewers using the Physiotherapy Evidence Database (PEDro) scale, a tool comprised of 11 items (**Table 3**) that evaluates four methodological domains: randomization, blinding, group comparison, and data analysis (26). The scoring system is based on a model of a Yes (1 point) or No (0 point) response rating scale (27). Each study's score was derived by adding the final scores obtained, as qualifying criteria were excluded from the total score due to their relevance to external validity. The PEDro scale measures methodological quality on a scale of 0–10, with a higher PEDro score indicating higher methodological quality: 8–10 indicates outstanding methodological quality; 5–7 indicates good methodological quality; 3–4 indicates medium methodological quality; and <3 indicates poor methodological quality (28). Additionally, this analysis made use of best evidence synthesis (BES) to assess the entire body of scientific data (29). The BES divides evidence into five categories depending on the methodological quality, the quantity of research, and the consistency of their findings (30). (1) Strong evidence: more than two high-quality studies concurred on the findings; (2) moderate evidence: one high-quality study and numerous low-quality studies concurred on the findings; (3) limited evidence from a single study or inconsistent conclusions from numerous studies; (4) conflicting evidence: there are conflicting study findings, while 75% of studies revealed consistent findings; and (5) there is no evidence: this finding was not made in any study.

## Results

### Study Selection

Figure 1 illustrates the selection of records. We identified 179 papers in the initial search and six additional articles via manual searching. After reviewing the titles of 185 studies, 46 were excluded. We removed an additional 124 studies from the remaining 133 based on the exclusion criteria. Thus, nine RCTs and non-RCTs were included in this review of the literature to assess the effect of exercise training on the physical fitness of young tennis players (Table 2).

**Table 2:** Characteristics of included studies.

**References**	**Design**	**Population characteristics**	**Interventions**	**Type of exercise training**	**Measures index**	**Outcomes**
Fernandez-Fernandez et al. (17)	Pre-post test	Sex: M, EG: *n* = 8, CG: *n* = 8, age: 16.9 ± 0.5 yr., WT: 74.7 ± 5.3 kg, ht.: 1.80 ± 3.6 m, TB: 8.0 ± 2.6 yr. DH: NP	Freq.: 2 times/week, time: 60 min, Length: 8 weeks	Combined explosive strength and repeated sprint-training (EG1), Control group (CG)	Speed (10, 20, 30m, RAS), Power (VJ)	10 m↑, RSA↑, 20 m →, 30 m →, VJ↑
Fernandez-Fernandez et al. (18)	Pre-post test	*n* = 60, Sex: male, TB: NP; age: 12.5 ± 0.3 yr., WT: 44.2 ± 7.0 kg, ht.: 156.6 ± 7.08 cm, DH: RH = 48, LH = 12	Freq.: 2 times/week, time: 30–60 min, Length: 8 weeks	Plyometric training (EG1), Control group (CG)	Speed (20 m sprint test), Agility (505 test), Power (CMJ), Strength (SLJ and OMBT)	20 m↑, 505 test↑, CMJ↑, SLJ↑, OMBT↑
Fernandez-Fernandez et al. (19)	Pre-post test	*n* = 20, Sex: NR, TB: 6 ± 1.2 yr., EG: age = 14.8 ± 0.1 yr., WT = 63.8 ± 7.1 kg, ht. = 174.7 ± 4.8 cm, DH: RH = 16; LH = 4	Freq.: 2 times/week, time: 40 min, Length: 8 weeks	Mixed high-intensity intermittent runs and Tennis- specific training (EG1), Tennis-specific drills only (CG)	Speed (20 m dash), Agility (505 test), Power (VJ)	20 m →, 505 test↑, VJ →
Fernandez-Fernandez et al. (20)	Pre-post test	*n* = 16, age: 12.9 ± 0.4 yr., WT: 46.0 ± 5.7 kg, ht.: 157.0 ± 5.1 cm, TB: 3.0 ± 1.2 yr., Sex: M; DH: RH = 13, LH = 3	Freq.: 2 times/week, time: 32.4 ± 7.3 min, Length: 5 weeks	Neuromuscular training before tennis specific training (EX1), conducted Neuromuscular training after tennis specific training (EG2)	Speed (5, 10, 20m), Agility Test (505 test), Power (CMJ), Strength (OMBT)	EG1: 5 m↑, 10 m↑, 20 m↑, 505 test↑, CMJ↑, OMBT↑, EG2:10 m↑, 20 m↓, 5 m↓, 505 test↓, CMJ↓, OMBT →
Fozia et al. (21)	Pre-post test	*n* = 30, Sex: NR; TB ≥ 1 yr., EG: age = 15.20 ± 0.41 yr., BMI = 20.23 ± 1.54, CG: age = 15.53 ± 1.06 yr., BMI = 20.71 ± 1.53, DH: NP	Freq.: 3 time/week, time: NR, Length: 5 weeks	Core training (EG1), Control group (CG)	Agility (*T*-test)	*T*-test↑
Kilit et al. (22)	Pre-post test	Sex: M, TB ≥ 2 yr., EG1: *n* = 13, age: 13.5 ± 0.2 yr., ht.: 151 ± 5 cm, WT: 45.5 ± 4.2 kg, EG2: *n* = 13, age: 13.3 ± 0.3 yr., ht.: 153 ± 3 cm, WT: 46.9 ± 4.8 kg, DH: NP	Freq.: 3–4 times/week, time: 80 min, Length: 4 weeks	Good performers group (EG1), Moderate performers group (EG2)	Speed (10, 20 m sprint), agility (T-drill test)	10 m↑, 20 m↑, T-drill test↑
Kilit and Arslan (23)	Pre-post test	*n* = 29, Sex: M, age: 13.8 ± 0.4 yr., ht.: 159.2 ± 7.5 cm, WT: 49.4 ± 6.1 kg, TB ≥ 2 yr., DH: RH = 29	Freq.: 3 times/week, time: 8–16min, Length: 6 weeks	High-intensity interval training (EG1), On court tennis training (EG2)	Speed (5, 10, 20, 400 m), Agility (T-drill test), Power (CMJ, SJ, DJ)	5 m↑, 10 m↑, 20 m↑, 400 m↑, T-drill test↑, CMJ↑, SJ↑, DJ↑
Moya-Ramon et al. (24)	Pre-post test	Sex: M, TB = 9.0 ± 2.6 yr., EG1, *n* = 10; Age: 16.7 ± 0.1 yr., WT: 72.0 ± 5.2 kg, ht.: 181.6 ± 4.8 cm, CG, *n* = 10; Age: 16.4 ± 0.3 yr., WT: 71.1 ± 7.2 kg, ht.: 179.9 ± 4.4 cm, DH: NP	Freq.: 2 times/week, time: NR, Length: 6 weeks	Resisted Sprint Training (EG1), Conventional Sprint Training (CG)	Speed (5, 10, 20 m sprint, and RSA), Agility (505 test), Power (VJ), Strength (SLJ)	5 m↑, 10 m →, 20 m →, RSA →, 505 test →, VJ↑, SLJ↑
Santos-Rosa et al. (25)	Pre-post test	TB: 5.0 ± 1.2 yr., Sex: NP, EG1: *n* = 14, age: 14.96 ± 0.88 yr., WT: 60.34 ± 9.13 kg, ht.: 172.50 ± 7.08 cm, EG2: *n* = 15; age: 15.21 ± 1.40 yr., WT: 59.50 ± 10.90 kg, ht.: 172.57 ± 7.90 cm, DH: RH = 27, LH = 2	Freq.: 3 times/week, time: 75.5 ± 6.4 min, Length: 8 weeks	Neuromuscular Warm-up after tennis-specific training (EG1), Dynamic Warm-up before tennis-specific training (EG2)	Speed (5, 10, 20 m), Agility (505 test), Power (VJ), Strength (BMBT, FMBT, OMBT)	5 m↑, 10 m↑, 20 m↑, 505 test →, VJ↑, FMBT↑, OMBT↑, BMBT↓

*DH, dominant hand; WT, weight; ht., height; NR, not reported; yr., year; M, male; F, female; Freq., frequency; CG, control group; EG, experimental group; RH, right-handed; LH, left-handed; CMJ, vertical countermovement jump; SLJ, standing long jump; RSA, repeated sprint shuttle test; 505 test, modified 505 agility test; SJ, squat jumping; DJ, drop jumping; VJ, vertical jump; OMBT, overhead medicine ball throw; FMBT, forehand medicine ball throw; BMBT, backhand medicine ball throw*.

### Study Quality Assessment

The Physiotherapy Evidence Database scores varied from 4 to 7 for the studies included in this review (Table 3). There were eight publications with a PEDro score of > 5, and only one with a PEDro score of 4, indicating that the included studies had a high degree of methodological quality and that the research findings were reasonably reliable. Additionally, all of these studies satisfied the criteria for randomization, group similarity at baseline, between-group comparison processes, point measurements, and variability; seven studies met the criteria for intention to treat analysis; and only one study met the blind assessor criterion. However, no study has been conducted to justify the use of concealed allocation concealment, blind participants, or blind therapists.

**Table 3:** Methodological quality assessment.

**References**	**Fernandez-Fernandez et al. (17)**	**Fernandez-Fernandez et al. (18)**	**Fernandez-Fernandez et al. (19)**	**Fernandez-Fernandez et al. (20)**	**Fozia et al. (21)**	**Kilit et al. (22)**	**Kilit and Arslan (23)**	**Moya-Ramon et al. (24)**	**Santos-Rosa et al. (25)**
Eligibility criteria	1	1	1	1	1	1	1	1	1
Random allocation	1	1	1	1	1	1	1	1	1
Allocation concealment	0	0	0	0	0	0	0	0	0
Group similar at baseline	1	1	1	1	1	1	1	1	1
Blind subject	0	0	0	0	0	0	0	0	0
Blind therapist	0	0	0	0	0	0	0	0	0
Blind assessor	0	0	1	0	0	0	0	0	0
Follow-up	1	1	1	1	1	0	1	0	1
Intention to treat analysis	1	1	1	1	1	0	1	1	0
Between group comparisons	1	1	1	1	1	1	1	1	1
Point measure and variability	1	1	1	1	1	1	1	1	1
**PEDro total score**	6	6	7	6	6	4	6	5	5

### Population Characteristics

The population characteristics of the nine studies included in this review were evaluated on the following aspects: (1) sample size: there were 246 participants in the nine studies, ranging from 16 to 60, with a median of 26 and a mean of 27.3 sample size; (2) gender: all nine of these studies examined young tennis players. Six research studies focused exclusively on men, whereas the remaining three studies did not specify gender; (3) age: none were over the age of 18. The age varied from 12.2 to 17.1 years, with a median of 13.8 and a mean of 14.2 years; (4) training background: eight studies reported the training background of the participants; (5) dominant hand: five studies reported on the dominant hand of participants, while four articles did not report on the dominant hand of participants.

### Interventions Characteristics

In total, 13 intervention programs were utilized in the included studies (Table 2). These interventions included resisted training (24), core training (21), a combination of high-intensity intermittent rounds and tennis-specific training, tennis-specific exercises (19), plyometric training (18), a combination of explosive strength and repeated sprint training (17), neuromuscular warm-up (NWU) training, dynamic warm-up (DWU) training (25), neuromuscular training (NMT) prior to tennis-specific training, NMT post-tennis-specific training (20), various stretching exercises (22), high-intensity interval training (HIIT), and on-court tennis training (OTT) (23). The duration of the trials covered in the nine studies ranged from 4 to 8 weeks (mean 6.6 weeks). Additionally, the majority of studies used one to three training sessions per week and varied the time of each session from 30 to 90 min.

All of the studies were RCTs with a pre-post design. Four studies had an experimental (EG) and a control group (CG) (17, 18, 21, 24), while only five studies included two experimental and no CGs (19, 22, 23, 25).

### Outcome and Measures

#### Effect of Exercise Training on Speed

Among the included literature, eight studies examined speed. According to one study (22), when compared to static and static-dynamic stretching protocols, dynamic and static + dynamic stretching procedures resulted in a significant difference in 10 m acceleration and 20 m sprint time (*p* < 0.05). Additionally, when compared to static, static + dynamic, and dynamic + static stretching procedures, dynamic and non-stretching approaches resulted in a significant difference in performance between good and relatively mediocre performers (*p* < 0.05). Santos-Rosa et al. (25) found that when compared to DWU training, an NWU significantly improved sprint (5, 10, and 20 m) performance (*p* < 0.05). Fernandez-Fernandez et al. (18) reported that combining plyometric exercise with traditional tennis training has a significant (*p* < 0.01) effect on speed (20 m sprint test). Another study found that HIIT outperformed OTT in terms of speed (5, 10, 20, 400 m sprint) (23). Within the group, significant changes in sprinting (5, 10, and 20 m sprints) and the 400 m running test time were observed between pre- and post-testing in the HIIT and OTT sessions (*p* < 0.05) (23).

Four studies concluded that certain speed indicators were statistically significant following exercise training. In the study conducted by Fernandez-Fernandez et al. (17), they observed a statistically significant (*p* < 0.05) improvement in speed (10 m sprint test) and a highly significant (*p* < 0.01) improvement in repeated sprint ability, but there were no significant changes in the sprint 20 and 30 m test (17). Moya-Ramon et al. (24) compared EG and CG using a resisted sprint training program. They concluded that while speed on the 5 m sprint time was considerably increasing (effect size 0.29), no significant difference was observed in the 10 m sprint time, 20 m sprint time, or repeated-sprint ability shuttle time (effect size −0.03).

Fernandez-Fernandez et al. (20) found that the NMT before tennis-specific training group had a positive effect on pre- to-post-test measures of speed (5 m—effect size 0.52; 10 m—effect size 0.32; and 20 m—effect size 1.08), whereas the NMT after tennis-specific training group had trivial effects on 10, 20 m, or negative effects on 5 m. Similarly, Fernandez-Fernandez et al. (19) reported that combining high-intensity training with sport-specific drill sessions vs. only sport-specific drill sessions resulted in no significant improvement in speed (20 m dash).

#### Effect of Exercise Training on Agility

In eight of the studies included, the agility test was examined. Fernandez-Fernandez et al. (18) found that combining plyometric training with regular tennis training had a statistically significant (*p* < 0.01) effect on agility (modified 505 agility test). Kilit et al. (22) showed that dynamic and general static + dynamic stretching methods improved agility (T-drill test) significantly more than static and static + dynamic stretching sessions (*p* < 0.05). Additionally, dynamic and non-stretching approaches indicated a significant difference in performance between excellent and generally mediocre performers compared to static, static + dynamic, and dynamic + static stretching sessions (*p* < 0.05) (22). Kilit and Arslan (23) demonstrate that HIIT vs. OTT significantly improved agility performance (T-drill agility test; *p* < 0.05). Fernandez-Fernandez et al. (19) found that combining high-intensity training and sport-specific drill sessions had a statistically significant benefit on agility (505 agility test) when compared to solely sport-specific drill training. Fozia et al. (21) assessed the effects of core training on agility and the intervention group had a substantial improvement (*p* = 0.001) when compared to the CG.

Three studies found no significant difference in agility between the groups. Santos-Rosa et al. (25) compared an NWU to a DWU and found no significant improvement in agility (modified 5-0-5 change in direction evaluation; *p* > 0.05). Moya-Ramon et al. (24) examined the EG and CG using a resistant vs. normal sprint training program. The data indicate that agility, as measured by the modified 5-0-5 agility assessment (dominant and non-dominant limb), did not improve. In another study (20), the NMT before tennis-specific training group showed favorable benefits on agility (effect size 0.22), while the NMT after tennis-specific training group demonstrated negative effects on the modified 5-0-5 agility test (effect size −0.24).

#### Effect of Exercise Training on Power

In seven of the studies included, a speed test was examined. Kilit and Arslan (23) published results on HIIT vs. OTT; both training protocols significantly improved jumping performance (countermovement jumping, squat jumping, and drop jumping) from pre- to post-testing (*p* < 0.05). Moya-Ramon et al. (24) compared EG and CG using a resistant sprint training program. The results indicate that power (as measured by the vertical jump test) was significantly enhanced (*p* < 0.05). Fernandez-Fernandez et al. (17) revealed that combining explosive strength with repeated sprint training sessions resulted in a statistically significant (*p* < 0.05) increase in vertical jumping power. Fernandez-Fernandez et al. (18) further found that combining plyometric exercise with traditional tennis training resulted in a statistically significant increase (*p* < 0.01) in power (vertical countermovement jump). Santos-Rosa et al. (25) found that NWU was associated with a statistically significant (*p* < 0.05) increase in power when compared to a DWU training program (vertical jumping). Fernandez-Fernandez et al. (19) observed no significant gain in power following a training intervention combining high-intensity training and sport-specific training (vertical jumping). There was a considerable increase in power (effect size 0.29) in the NMT before the tennis particular training group, but only a minor decrease in countermovement (effect size −0.03) in the NMT after tennis-specific training group was found (20).

#### Effect of Exercise Training on Strength

In four of the studies included, the strength test was examined. Fernandez-Fernandez et al. (18) adopted an intervention program that included plyometric exercise and regular tennis training. They found a statistically significant (*p* < 0.01) improvement in standing long jump test and overhead medicine ball throw. Moya-Ramon et al. (24) compared EG and CG using a resistant vs. normal sprint training program. They found that standing long jump strength was significantly improved (effect size 0.31).

Two studies concluded that there was no statistically significant difference in strength. Santos-Rosa et al. (25) found that NWU sessions had no effect on the backhand medicine ball throw test (*p* > 0.05), but had an effect on the forehand medicine ball throw test (*p* = 0.004) and overhead medicine ball throw test (*p* = 0.014). Another study conducted by Fernandez-Fernandez et al. (20) showed that the NMT before tennis-specific training group achieved favorable improvements in terms of strength (effect size 0.51) and the trivial overhead medicine ball throw (effect size 0.02).

#### Effect of Exercise Training on Flexibility

Flexibility was evaluated only in one study (25), which examined flexibility using the shoulder range of motion test (external rotation range of motion and total range of motion). Flexibility tests reveal that the NWU significantly increased (*p* < 0.05) shoulder external rotation range of motion and overall range of motion on the dominant side.

## Discussion

The purpose of this study was to examine the effects of exercise training interventions on the physical fitness components of young tennis players and to determine whether exercise training can have a beneficial effect on these components. Nine studies' preliminary data revealed substantial evidence that exercise training enhanced athletes' physical fitness in terms of speed, agility, strength, and flexibility. Meanwhile, there is conflicting evidence regarding the effect on power, and yet there is no evidence regarding the effect of exercise training on endurance. These studies' conclusions show the beneficial effect of exercise training on young tennis players. However, the methodological quality of these studies varied, and future research should aim to improve methodological quality in order to better explain the effect of exercise training on physical fitness.

### Effect of Exercise Training on Speed

Running skills of the tennis players directly affect whether a game wins or loses (31). Tennis players run an average of 3 m each time they strike the ball. Athletes must run between 8 and 12 m to earn a ball score, which establishes a high threshold for athletes' movement speed. Notably, the majority of the research examined (*n* = 8) have concentrated on speed (strong evidence). Tennis players' speed was assessed using sprint test (20 m linear sprint test), repeated sprint shuttle test (RSA) (17, 24), 20 m dash (with 5 and 10 m split times) (18–20, 23, 25), 10 m sprint (17), 20 m (17, 22), 30 m sprint (17), and 400 m running test (23). The data indicated that resisted training (24), mixed high-intensity intermittent running sessions, tennis-specific training, tennis-specific drills (19), plyometric training (18), combined explosive strength and repeated sprint training (17), NWU, DWU training (25), NMT prior to tennis specified training, NMT post-tennis specified training (20), different stretching training (22), HITT, and OTT training (23) can help tennis players to improve their speed. Specifically, the four studies showed a statistically significant effect of exercise training on speed using various stretching programs (22), NWU vs. DWU training (25), plyometric training combined with regular tennis training (18), and HIIT vs. OTT training (22). Other studies examined the effect of their training programs on speed using a combination of explosive strength and repeated sprint training (17), resisted sprint training (24), NMT before or after a tennis session, combining HIIT and sport-specific drills vs. only sport-specific drill training (19) and found no statistically significant effect of their training methods on speed.

### Effect of Exercise Training on Agility

Agility is a critical component of physical fitness (32), since it enables tennis players to quickly shift their direction of movement (33). It can be divided into three distinct categories: forward occurs approximately 47% of the time; sideways occurs ~48% of the time; and backward occurs around 5% of the time (31). In the eight studies that reported on agility (strong evidence), agility was assessed using a modified variant (i.e., stationary start) of the 505 agility test (18–20, 24, 25) and T-drill test or T-test (21–23). The data showed that resisted training (24), core training (21), mixed high-intensity intermittent running sessions and tennis-specific training, tennis-specific drills (19), plyometric training (18), NWU training, DWU training (25), NMT (20), different stretching training (22), and HIIT vs. OTT (23) can help tennis players to improve their agility. Specifically, the five studies demonstrated a statistically significant effect of exercise training on agility when plyometric training was combined with traditional tennis training (18), different stretching programs were used (22), HIIT vs. OTT was used (23), combining HIIT and sport-specific drill sessions was used instead of sport-specific drill training alone (21), and core training was used (21). The other studies compared NWU to a DWU training program (25), resisted sprint training to a conventional sprint training program (24), and NMT before or after a tennis session training program (20) and found no statistically significant effect of their training programs on agility.

### Effect of Exercise Training on Power

Power is a subset of speed power, which is the foundation for numerous sports (34). Not only can it overcome resistance and cause the object to produce displacement, but it may also cause the object to produce a huge displacement speed (35). Seven studies (conflicting evidence) evaluated power using the vertical jump test (countermovement jumping) (17–20, 22, 24, 25). Only one study, however, included three vertical jump tests: countermovement jumping, squat jumping, and drop jumping (23). The types of exercises that can improve the power of young tennis players included resisted training (24), mixed high-intensity intermittent running sessions and tennis-specific training sessions, tennis-specific drills (19), plyometric training (18), combined explosive strength and repeated sprint training (17), NWU training, DWU training (25), NMT (20), and HIIT vs. OTT (23). Specifically, six studies indicated a statistically significant effect of exercise training on power using an HIIT vs. OTT protocol (23), a resisted sprint training program (17), a plyometric training program in combination with a regular tennis training program (18), and an NWU (25). The other studies used a combination of HIIT and sport-specific drill training sessions vs. only sport-specific drill training sessions alone (19), or NMT before or after a tennis session program (20) and found that their training programs had no statistically significant effect on power, but the NMT before tennis-specific training group achieved a positive effect from pre-test to post-test measures in power.

### Effect of Exercise Training on Strength

Strength is the foundation for all other components of physical fitness (e.g., speed and endurance) (36). However, just four studies on strength have been published (limit evidence). Following the standing long jump test, the medicine ball throw, and the shoulder strength test, strength was assessed. However, there is only one study that evaluated strength using both the overhead medicine ball throw and the standing long jump (18). One study passed the shoulder strength test and the medicine ball throw test (i.e., overhead, forehand, backhand, and forehand and backhand) (25). The types of exercise training used to improve strength were resisted training (24), plyometric training (18), NWU training, DWU training (25), and NMT (20). Our analysis of the literature revealed that different types of exercise training have varying training effects. Specifically, two studies demonstrated a statistically significant effect of exercise training on strength when a plyometric training program was combined with regular tennis training (18) and when a resisted sprint training program was used (24). The other studies compared NWU to a DWU training program (25), and NMT before or after a tennis session program (20) and found that their training programs had no statistically significant effect on strength. These findings may be explained by the fact that growth and maturation can be connected with these increases in strength and power, as it has been indicated that adolescents should undergo a performance spurt in strength and power following the onset of puberty (37).

### Effect of Exercise Training on Flexibility

Athletes should place a premium on flexibility development during training, as flexibility enhances muscle strength and movement range (38). However, just one study has examined the subject of flexibility (limit evidence). The results reveal that both groups, NWU and DWU, improved their shoulder external rotation and overall range of motion significantly. However, incorporating an NWU with a lower volume (i.e., 20–35 min) that involves general mobility, core, and shoulder strength routines in conjunction with neuromuscular-related exercises, such as plyometric and acceleration/deceleration/COD drills, has been shown to significantly improve the overall flexibility training of young tennis players (25). This study compared solely the NWU and DWU interventions (25); it made no comparisons to other forms of exercise training. As a result, additional research is required in the future to focus on flexibility.

### Limitations

This systematic review has a number of limitations. To begin, there are no reports of endurance in the research included. Endurance is a critical component of physical fitness and a vital indicator of a tennis player's physical fitness (39). However, a lack of research on this physical fitness component may impair the study's completeness or create loopholes in the examination of athletes' physical fitness components. Second, three of the studies did not include information about the athletes' gender. If it exists, it may be significant because gender is a significant influencing factor when judging young tennis players' physical fitness. At the same time, there is no report on female athletes, suggesting that future studies should include female athletes. Thirdly, this review chose to include only English-language papers, which indicates that some pertinent empirical material may be omitted. Fourthly, this study focused exclusively on six components of physical fitness, omitting the effect of exercise training on other components of physical fitness. This is because the International Tennis Federation considers that the six physical fitness components provide complete representations of a tennis player's physical fitness level (9).

## Conclusion

The review found compelling evidence that exercise training increased the physical fitness of young tennis players in terms of speed and agility, but there was insufficient evidence for strength and flexibility. Meanwhile, there is conflicting evidence regarding the effect of power, but no evidence regarding the effect of exercise training on endurance was found. These findings may give tennis practitioners with insight into the importance of considering the interplay between physical fitness components when measuring and developing talent, rather than focusing exclusively on the performance of a single physical fitness component. Additionally, there is a lack of studies in the research field that examines the relationship between exercise training and physical fitness in female tennis players. To gain a better understanding of the relationship and effect of various exercise training methods on the multifactorial nature of physical fitness performance in young tennis players, high-quality, comprehensive evidence is required, covering athletes of various sports levels.

## Data Availability Statement

The original contributions presented in the study are included in the article/supplementary material, further inquiries can be directed to the corresponding authors.

## Author Contributions

The literature search, study selection, and study quality assessment were completed by WX and XB, and SG arbitrated the study to include any disagreements. OT, MN, and CZ participated in the review of the manuscript. TB and WL participated in the revision and reading of the manuscript. All the authors made contributions to the article and reviewed the submitted manuscript.

## Conflict of Interest

The authors declare that the research was conducted in the absence of any commercial or financial relationships that could be construed as a potential conflict of interest.

## Publisher's Note

All claims expressed in this article are solely those of the authors and do not necessarily represent those of their affiliated organizations, or those of the publisher, the editors and the reviewers. Any product that may be evaluated in this article, or claim that may be made by its manufacturer, is not guaranteed or endorsed by the publisher.
